# Synchronous Gastric and Colonic Adenocarcinoma: A Case Report With its Molecular Implications

**DOI:** 10.7759/cureus.56607

**Published:** 2024-03-20

**Authors:** Lakshmipriya V, Sarah Grace Priyadarshini, Neha Agarwal, Padmapriya B S

**Affiliations:** 1 Department of Pathology, Saveetha Medical College and Hospital, Saveetha Institute of Medical and Technical Sciences, Saveetha University, Chennai, IND

**Keywords:** microsatellite instability., mismatch repair, colonic cancer, gastric cancer, synchronous neoplasms

## Abstract

Multiple primary tumors are rare but their incidence is increasing nowadays with advancements in diagnostic methods and the extended survival of individuals previously treated for malignancies. However, synchronous occurrence of gastric cancer (GC) and colonic cancer (CC) is a rare entity. A 41-year-old male came with complaints of epigastric pain associated with anorexia, rapid weight loss, and occasional constipation. Contrast-enhanced computed tomography (CECT) of the abdomen and pelvis reported mucosal thickening in the antrum, likely GC with circumferential wall thickening of the transverse colon with pericolic fat stranding suggestive of CC. Upper gastrointestinal endoscopy and colonoscopy were also done and a biopsy was taken from representative sites, which confirmed malignancy. He completed three cycles of chemotherapy preoperatively and underwent subtotal gastrectomy, D2 lymphadenectomy, gastrojejunostomy, jejunojejunostomy, and transverse colectomy simultaneously. Histopathological examination confirmed moderately differentiated gastric adenocarcinoma penetrating into the subserosa and well-differentiated colonic adenocarcinoma invading the muscularis propria. Immunohistochemical analysis of mismatch repair (MMR) proteins was done to determine the association with hereditary nonpolyposis colorectal cancer syndrome (HNPCC) or Lynch syndrome. The patient underwent postoperative chemotherapy along with immunotherapy. To conclude, synchronous occurrence of primary GC and primary CC with similar MMR protein expression in immunohistochemistry is an uncommon entity.

## Introduction

The term "multiple primary neoplasm," initially coined by Billroth in 1889, refers to the occurrence of multiple neoplasms in a single patient. However, only in 1932, with the extensive examination of 1,259 case reports conducted by Warren and Gates, the concept of multiple primary neoplasia gained significant recognition and attention [1]. The incidence of multiple primary malignant neoplasms rises with advancing age, partly attributed to advancements in diagnostic methods and the extended survival of individuals previously treated for malignancies. As reported in the literature, the overall occurrence rate of multiple primary malignant tumors is estimated to range from 0.73% to 11.7%. These are characterized as the presence of two or more distinct synchronous or metachronous cancers within the same or different systems [2]. Encountering two or more simultaneous primary malignancies in different digestive organs is a relatively uncommon occurrence in routine surgical practice. Multiple primary tumors are categorized into two groups: synchronous and metachronous. A tumor is labeled as synchronous if it is identified within six months of the first tumor, and it is termed metachronous if it is discovered more than six months after the initial tumor. Instances of synchronous cancer combinations include esophageal cancer and gastric cancer (GC), GC and colorectal cancer (CRC), and GC and duodenal cancer. However, the occurrence of simultaneous GC and colonic cancer (CC) is infrequent [3]. The occurrence of GC along with a second primary cancer ranges from 2.0% to 10.9% [1]. CRC is the most commonly observed cancer occurring simultaneously in individuals with GC with a frequency that varies between 20.1% and 37.2%. Identifying synchronous and metachronous cancers offers the chance to concurrently treat both malignancies through minimally invasive methods, which can have a positive impact on the patient's prognosis and overall quality of life [4]. 

Determining the molecular subtypes of CRC holds significant clinical relevance. One criterion for classifying CRC involves assessing the expression patterns of mismatch repair (MMR) proteins. These nuclear enzymes play a crucial role in repairing base-base mismatches that occur during deoxyribonucleic acid (DNA) replication in proliferating cells. MMR proteins form complexes (heterodimers) that bind to regions of abnormal DNA, initiating their removal. The absence of MMR proteins results in the accumulation of DNA replication errors, particularly in genomic regions with short repetitive nucleotide sequences-a phenomenon referred to as microsatellite instability (MSI). MSI is detectable in over 90% of CRC in patients with Lynch syndrome, while in sporadic CRC, it occurs in 15% of cases [5]. We present a case report detailing a rare occurrence of synchronous GC and CC and also its MSI evaluation. 

## Case presentation

**Figure 1:**
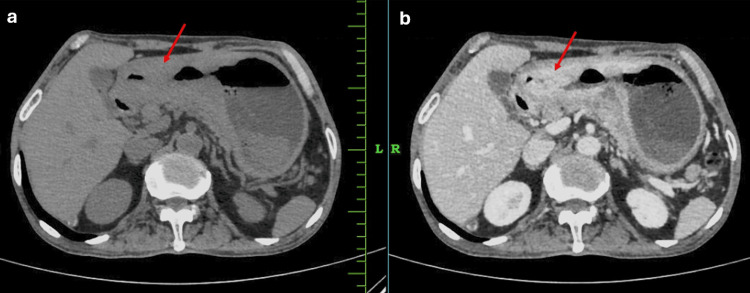
CT (a) plain and (b) contrast-enhanced (venous phase) images of the abdomen showing heterogeneously enhancing irregular asymmetrical circumferential mural thickening involving the antrum and pylorus of the stomach (red arrows) causing significant luminal narrowing.

**Figure 2:**
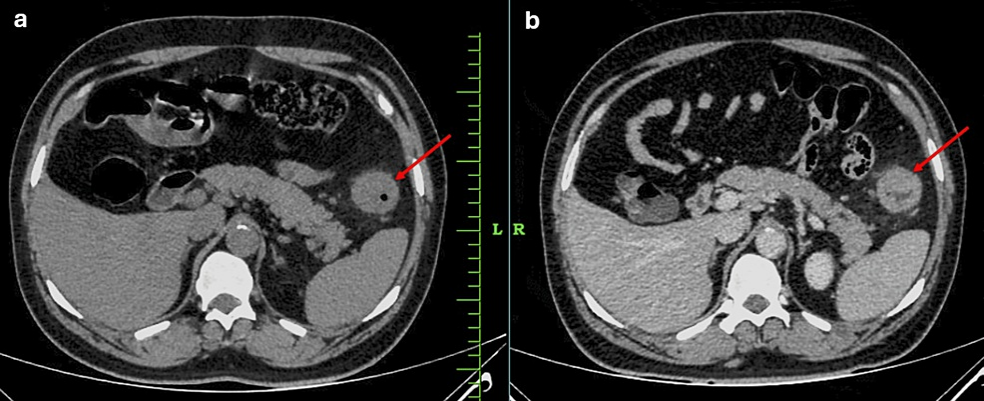
CT (a) plain and (b) contrast-enhanced (venous phase) images of the abdomen showing heterogeneously enhancing asymmetrical circumferential irregular wall thickening causing severe luminal narrowing involving splenic flexure (red arrows) with periserosal fat stranding.

A 41-year-old male, a nonsmoker and a diabetic for the past two years, was admitted to our institution with complaints of epigastric pain associated with anorexia, rapid weight loss, and occasional constipation. The patient’s past medical and family history was unremarkable. Complete blood count showed: a white blood cell count of 7160 cells/cu mm, haemoglobin 7.2g/dl, and platelet count of 3.36 lakhs/cu mm. Other laboratory findings were within their normal limits including tumor markers like serum carcinoembryonic antigen, cancer antigen 19-9, and α-fetoprotein. Contrast-enhanced computed tomography (CECT) of the abdomen and pelvis reported an irregular asymmetrical circumferential mural thickening involving the antrum and pylorus of the stomach, likely GC, as seen in Figure 1, with an asymmetrical circumferential irregular wall thickening causing severe luminal narrowing involving splenic flexure of transverse colon with periserosal fat stranding suggestive of carcinoma colon as seen in Figure 2.

Upper gastrointestinal endoscopy revealed an ulceroproliferative tumor in the antrum and pylorus of the stomach, the biopsy of which came out to be a moderately differentiated adenocarcinoma in histopathological examination (HPE). Colonoscopy reported a 3 cm ulcerative polypoidal lesion in the splenic flexure of the transverse colon with luminal narrowing, the biopsy of which came as well-differentiated adenocarcinoma. The patient was diagnosed with synchronous gastric and colon adenocarcinoma. He completed three cycles of chemotherapy preoperatively and underwent subtotal gastrectomy, D2 lymphadenectomy, gastrojejunostomy, jejunojejunostomy, and transverse colectomy simultaneously, and specimens were sent for HPE. Postoperative recovery was uneventful. 

HPE

**Figure 3:**
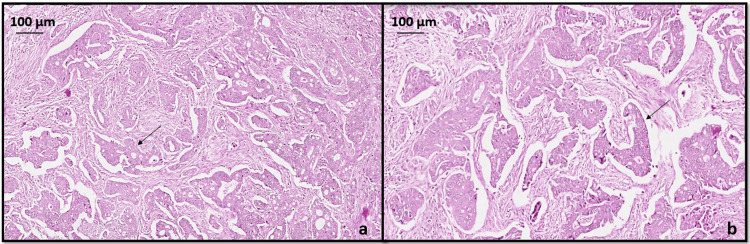
Hematoxylin and eosin stained sections (a) x40 and (b) x200 showing a moderately differentiated gastric adenocarcinoma, intestinal type (black arrow).

GC showed adenocarcinoma (ypT3ypN2) intestinal type (Lauren classification) with histopathological grade G2 moderately differentiated neoplasm penetrating into subserosal connective tissue without invasion of visceral peritoneum as seen in Figure 3. Lymphovascular invasion was seen without any perineural invasion. Four lymph nodes were positive for malignancy out of 25 lymph nodes examined in total. All margins were negative for invasive carcinoma.

**Figure 4:**
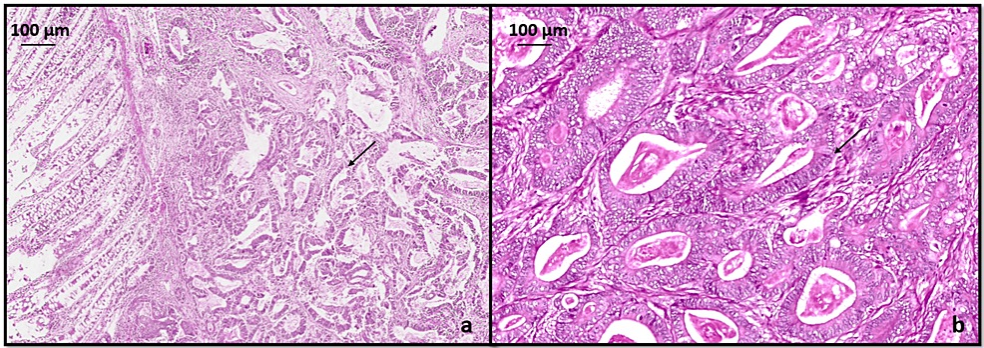
Hematoxylin and eosin stained sections (a) x40 (b) x400 showing a well-differentiated colonic adenocarcinoma (black arrow).

CC showed adenocarcinoma (ypT2ypN0) with histopathological grade G1 well differentiated as seen in Figure 4, invading into muscularis propria without any lymphovascular or perineural invasion and no lymph nodes were involved. All margins were negative for invasive carcinoma. The treatment effect was absent in both GC and CC with extensive residual cancer and no evidence of tumor regression (poor or no response, score 3) was seen.

**Figure 5:**
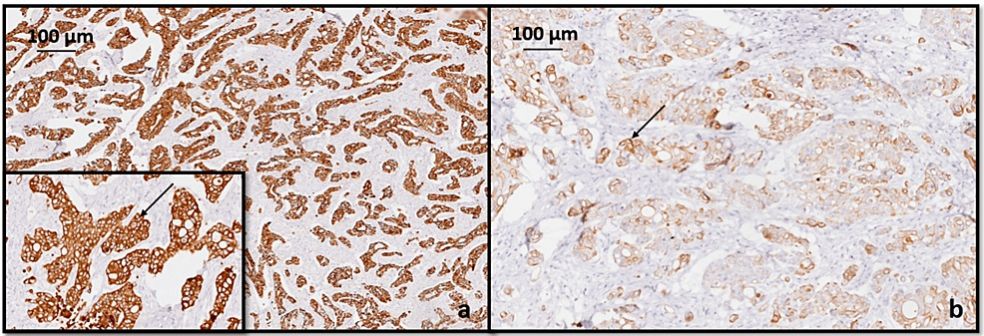
Immunohistochemical analysis of gastric carcinoma with (a) CK 7 showed strong cytoplasmic positivity (black arrow), (b) CK 20 showed moderate focal positivity (black arrow).

IHC analysis with CK 7 and CK 20 was done in both lesions. GC showed strong cytoplasmic positivity for CK 7 and moderate focal positivity for CK 20 as seen in Figure 5.

**Figure 6:**
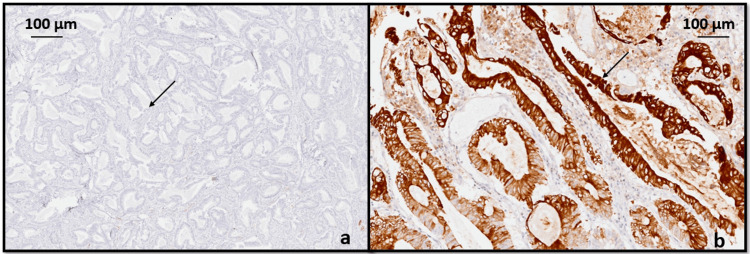
Immunohistochemical analysis for colonic carcinoma with (a) CK 7 is negative (black arrow), (b) CK 20 showed strong cytoplasmic positivity (black arrow).

CC showed strong cytoplasmic positivity for CK 20 and negative for CK 7 (Figure 6). From this pattern of staining we can conclude that it is a synchronous presentation of primary gastric and primary colonic adenocarcinoma.

**Figure 7:**
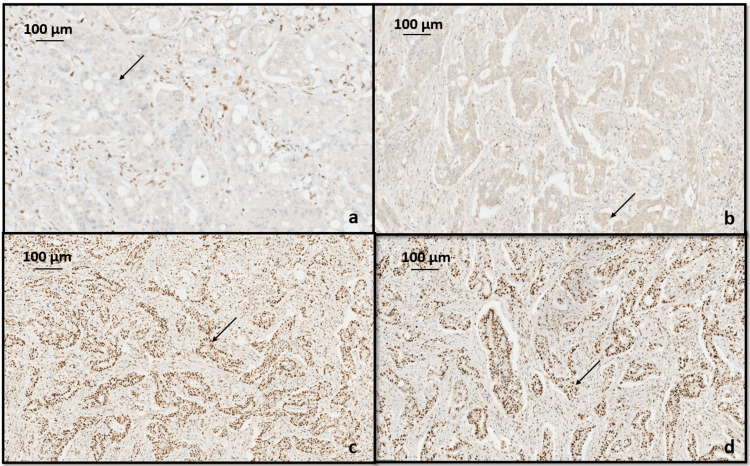
Immunohistochemistry for mismatch repair proteins in tumor cells of gastric adenocarcinoma showing (a) loss of nuclear expression of MLH1 (black arrow), (b) loss of nuclear expression of PMS2 (black arrow), (c) intact MSH2 nuclear expression (black arrow), (d) intact nuclear expression of MSH6 (black arrow).

**Figure 8:**
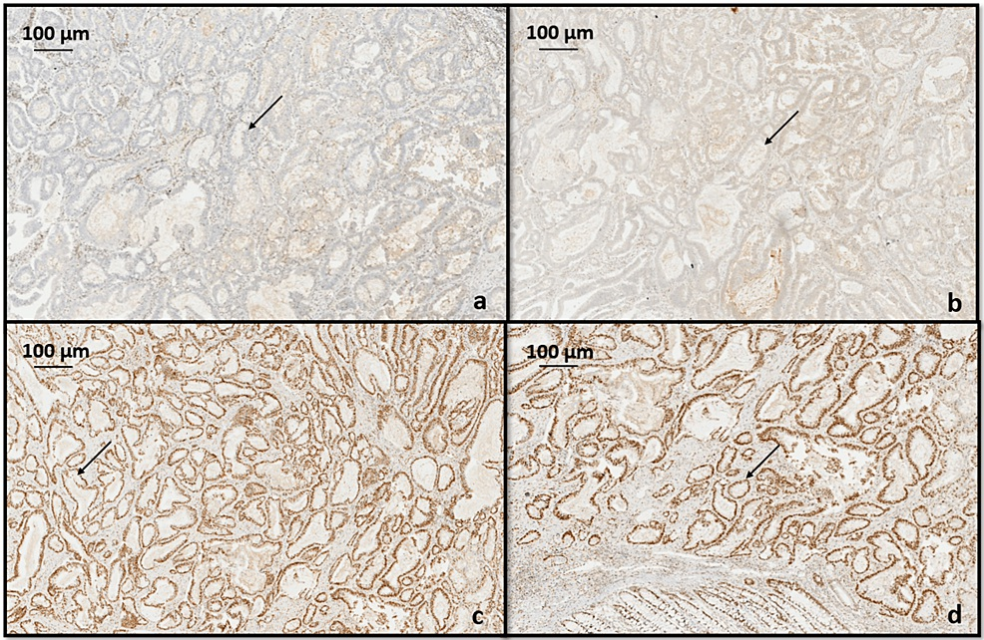
Immunohistochemistry for mismatch repair proteins in tumor cells of colonic adenocarcinoma showing (a) loss of nuclear expression of MLH1 (black arrow), (b) loss of nuclear expression of PMS2 (black arrow), (c) intact MSH2 nuclear expression (black arrow), (d) intact nuclear expression of MSH6 (black arrow).

Immunohistochemistry (IHC) analysis for MMR proteins including MLH1, MSH2, MSH6, and PMS2 was done to determine association with hereditary nonpolyposis colorectal cancer syndrome (HNPCC) or Lynch syndrome. In our case, both the gastric and colonic adenocarcinoma showed loss of nuclear staining for MLH1 and PMS2, whereas MSH2 and MSH6 showed intact nuclear staining in the tumor cells (Figures 7, 8).

The patient also received nine cycles of postoperative chemotherapy and immunotherapy and is currently stable without evidence of any metastasis.

## Discussion

With the extended survival rates for each cancer and more effective implementations of screening programs to detect early cancer lesions, a higher number of synchronous cancers will likely be identified. Among patients with GC, CRC is the most commonly found synchronous cancer, followed by cancers of the lung, esophagus, and liver in descending order of frequency. Most cases of CRC are identified before a diagnosis of GC. Among patients with both GC and CRC occurring simultaneously, the majority are at an early stage of GC [6]. The cause of synchronous cancer remains unidentified. Contributing factors encompass abnormalities in the DNA error repair mechanism, familial history, male gender, and advancing age (beyond 50 years) [3]. Warren and Gates initially established the definition of synchronous carcinoma: the lesions must be histopathologically confirmed as malignant, the lesions should be distinctly differentiated and locally isolated, and there should be an exclusion of the possibility that one of the tumors is metastatic [7]. 

As it is essential to distinguish between colonic metastasis from GC and simultaneous primary GC and CC, IHC staining with CK7 and CK20 can be performed. The identification of tumor cells exhibiting a CK7(−)/CK20(+) IHC staining pattern suggests primary CC. Conversely, a CK7(+)/CK20(−) staining pattern indicates primary GC [8]. Therefore, the diagnosis of synchronous GC and CC was made in the current case based on histological features and the results of IHC analysis. 

CC resulting from deficiencies in the DNA MMR system often exhibits MSI. This phenomenon is evident in both sporadic and more prevalent inherited cases of colorectal carcinoma. While polymerase chain reaction (PCR) amplification of microsatellite genes is the established gold standard for assessing microsatellite status, it is expensive, technically challenging, and not reliably feasible in routine pathology laboratories. IHC has been confirmed as an alternative method for mutation screening due to its sensitivity, accessibility, and cost-effectiveness [9]. In Lynch syndrome, a hereditary condition, the primary mechanism leading to the formation of a tumor with MSI involves a germline mutation in one allele of an MMR gene. This mutation combines with a somatic mutation to result in the functional loss of the corresponding non-mutant allele. Conversely, in sporadic colorectal cases, the abnormality in IHC commonly arises from aberrant methylation of the promoter region of the MLH1 gene. This methylation leads to transcriptional silencing, preventing the production of detectable protein [10]. Sporadic MSI cancers show methylation of MLH1 promoters; a condition, which is strongly correlated to the V600E mutation of the human gene BRAF (v-Raf murine sarcoma viral oncogene homolog B1) [11].

IHC could be employed to assess the expression of MMR proteins, aiming to identify the absence or loss of a specific protein within the tumor cell nucleus. An abnormal IHC pattern is strongly associated with MSI status as determined by PCR. MMR proteins are typically found in various human cells, especially in actively proliferating cells like crypt epithelium. The MMR proteins MLH1, PMS2, MSH2, and MSH6 naturally form heterodimer pairs in vivo, specifically MLH1/PMS2 and MSH2/MSH6. According to Chen et al., initially, immunostaining for MLH1, MSH2, MSH6, and PMS2 is done [12]. If all four proteins are present, no additional investigation is required unless specific clinical concerns arise. In cases where MLH1 and PMS2 are absent by IHC, the subsequent step involves analyzing BRAF either through PCR or IHC to determine whether the tumor is sporadic or necessitates further assessment for Lynch syndrome. PCR is preferred over IHC for BRAF analysis. If a BRAF V600E mutation is identified, no further testing is necessary, as the tumor is assumed to be a sporadic CRC. In the absence of a BRAF mutation, the next steps depend on the level of suspicion for Lynch syndrome based on the patient's age and medical history. If there is a high suspicion of Lynch syndrome, the tumor is submitted for genetic sequencing of MLH1 or PMS2. Conversely, if suspicion is low, an analysis of MLH1 promoter hypermethylation is conducted. If MLH1 promoter hypermethylation is not detected, the patient is then referred for sequencing of MLH1 or PMS2. Some laboratories opt for MLH1 methylation testing instead of BRAF mutational analysis after IHC in tumors showing the absence of MLH1 and PMS2. Similar to cases with BRAF mutations, those with MLH1 methylation are presumed to have sporadic tumors and do not require further molecular testing for LS [12]. In our case, there was a loss of MLH1 and PMS2 nuclear staining with intact MSH2 and MSH6 nuclear staining. Further investigations like BRAF mutation could be analyzed either through PCR or IHC, which was not done in our case.

The prognosis of patients with multiple tumors is predominantly influenced by the progression of GC, underscoring the importance of prioritizing GC surgery first. Additionally, solely performing colon surgery would hinder nutrient intake due to GC, while exclusive gastric surgery could lead to an anastomotic leakage caused by obstructive CC. Therefore, the preferred treatment approach, as illustrated in this case, involves simultaneous surgery to address both conditions [3]. Resection through D2 lymphadenectomy is the established standard treatment in oncology for GC and CRC. Consequently, simultaneous resection is recommended for all cases anticipating a curative outcome. Although radical oncologic surgery carries inherent risks of early and late postoperative complications, synchronous resection significantly raises the morbidity and mortality rates. The prevailing notion is that survival is directly influenced by the stage of GC and not by other synchronous carcinomas [7]. 

## Conclusions

Synchronous occurrence of primary GC and primary CC with similar MMR protein expression in IHC is an uncommon entity. Whenever feasible, a comprehensive surgical removal of both tumors should be performed simultaneously. It is crucial to differentiate between hereditary cases and sporadic cases with loss of MMR proteins, in order to implement the appropriate screening protocol for both the patients and their families. 
